# Time-course analysis and transcriptomic identification of key response strategies of *Nelumbo nucifera* to complete submergence

**DOI:** 10.1093/hr/uhac001

**Published:** 2022-02-11

**Authors:** Xianbao Deng, Dong Yang, Heng Sun, Juan Liu, Heyun Song, Yaqian Xiong, Yunmeng Wang, Junyu Ma, Minghua Zhang, Jing Li, Yanling Liu, Mei Yang

**Affiliations:** Key Laboratory of Plant Germplasm Enhancement and Specialty Agriculture, Wuhan Botanical Garden, Chinese Academy of Sciences, Wuhan 430074, China; Center of Economic Botany, Core Botanical Gardens, Chinese Academy of Sciences, Wuhan 430074, China; Key Laboratory of Plant Germplasm Enhancement and Specialty Agriculture, Wuhan Botanical Garden, Chinese Academy of Sciences, Wuhan 430074, China; Center of Economic Botany, Core Botanical Gardens, Chinese Academy of Sciences, Wuhan 430074, China; Key Laboratory of Plant Germplasm Enhancement and Specialty Agriculture, Wuhan Botanical Garden, Chinese Academy of Sciences, Wuhan 430074, China; Key Laboratory of Plant Germplasm Enhancement and Specialty Agriculture, Wuhan Botanical Garden, Chinese Academy of Sciences, Wuhan 430074, China; Key Laboratory of Plant Germplasm Enhancement and Specialty Agriculture, Wuhan Botanical Garden, Chinese Academy of Sciences, Wuhan 430074, China; University of Chinese Academy of Sciences, 19A Yuquanlu, Beijing, 100049, China; Key Laboratory of Plant Germplasm Enhancement and Specialty Agriculture, Wuhan Botanical Garden, Chinese Academy of Sciences, Wuhan 430074, China; University of Chinese Academy of Sciences, 19A Yuquanlu, Beijing, 100049, China; Key Laboratory of Plant Germplasm Enhancement and Specialty Agriculture, Wuhan Botanical Garden, Chinese Academy of Sciences, Wuhan 430074, China; University of Chinese Academy of Sciences, 19A Yuquanlu, Beijing, 100049, China; Key Laboratory of Plant Germplasm Enhancement and Specialty Agriculture, Wuhan Botanical Garden, Chinese Academy of Sciences, Wuhan 430074, China; University of Chinese Academy of Sciences, 19A Yuquanlu, Beijing, 100049, China; Key Laboratory of Plant Germplasm Enhancement and Specialty Agriculture, Wuhan Botanical Garden, Chinese Academy of Sciences, Wuhan 430074, China; University of Chinese Academy of Sciences, 19A Yuquanlu, Beijing, 100049, China; School of Chemistry, Chemical Engineering and Life Sciences, Wuhan University of Technology, Wuhan 430070, China; Key Laboratory of Plant Germplasm Enhancement and Specialty Agriculture, Wuhan Botanical Garden, Chinese Academy of Sciences, Wuhan 430074, China; Center of Economic Botany, Core Botanical Gardens, Chinese Academy of Sciences, Wuhan 430074, China; Key Laboratory of Plant Germplasm Enhancement and Specialty Agriculture, Wuhan Botanical Garden, Chinese Academy of Sciences, Wuhan 430074, China; Center of Economic Botany, Core Botanical Gardens, Chinese Academy of Sciences, Wuhan 430074, China

## Abstract

Water submergence is an environmental stress with detrimental effects on plant growth and survival. As a wetland plant species, lotus (*Nelumbo nucifera*) is widely cultivated in flood-prone lowlands throughout Asian countries, but little is known about its endurance and acclimation mechanisms to complete submergence. Here, we performed a time-course submergence experiment and an RNA-sequencing transcriptome analysis of the two lotus varieties “Qiuxing” and “China Antique”. Both varieties showed low submergence tolerance, with a median lethal time of approximately 10 days. Differentially expressed gene (DEG) analysis and weighted gene co-expression network analysis (WGCNA) identified a number of key genes putatively involved in lotus submergence responses. Lotus plants under complete submergence developed thinned leaves and elongated petioles containing a high density of aerenchyma. All four lotus submergence-responsive *ERF-VII* genes and gene sets corresponding to the low oxygen “escape” strategy (LOES) were elevated. In addition, a number of lotus innate immunity genes were rapidly induced by submergence, probably to confer resistance to possible pathogen infections. Our data also reveal the probable involvement of jasmonic acid in the modulation of lotus submergence responses, although to a lesser extent than the gaseous hormone ethylene. These results suggest that lotus plants primarily use the LOES strategy to cope with complex submergence-induced stresses, and they will be valuable for understanding the molecular basis underlying plant submergence acclimation.

## Introduction

Flooding is a major environmental stress that affects plant growth, crop productivity, and agricultural ecosystems. According to the data of the Food and Agriculture Organization (FAO) of the United Nations, floods have caused almost two-thirds of the global crop production loss between 2006 and 2016. [1] Flooding can be classified into waterlogging and submergence depending on water depth, with the former usually caused by heavy rain, when superficial water covers only plant roots, whereas the latter normally occurs during the rainy season, when water covers part or all aerial plant tissues [2, 3].

Partial or complete submergence substantially reduce the gaseous exchange between plants and their surrounding environment, limiting oxygen uptake and restricting aerobic respiration [4, 5]. Complete submergence also reduces light intensity, thus inhibiting underwater photosynthesis [6]. Under prolonged submergence, plants suffer low photosynthesis and are continuously compelled to take inefficient anaerobic metabolism, which causes energy crisis and nutrient deficiency, resulting in accumulation of phytotoxic end-products [7]. In addition, prolonged submergence and high humidity environment attenuates plant defense response system, increasing their susceptibility to diseases infection and pest invasion [8, 9]. Post flooding, submerged plants are suddenly exposed to oxygen-rich air, resulting in quick oxygen intake and overproduction of reactive oxygen species (ROS) [10, 11]. Therefore, these submergence-induced stresses require plants to undergo rapid adjustments to survive.

Plants have evolved two major adaptive strategies to cope with submergence-induced stress, including, a “quiescence” strategy based on *Submergence-1A* (*SUB1A*) and an “escape” strategy based on *SNORKEL1* (*SK1*) and *SNORKEL2* (*SK2*). In rice (*Oryza sativa*), the *SUB1* locus contains a cluster of two or three tandemly arranged Ethylene Response Factors (ERF). Most rice varieties contain *SUB1B* and *SUB1C* genes at the *SUB1* lotus, whereas only flood tolerant varieties contain the *SUB1A* gene [12, 13]. During submergence, *SUB1A* slackens the production of gibberellic acids (GA) and negatively regulates GA signaling, which inhibits the GA-mediated underwater growth, conserves energy, and enables prolonged endurance [5, 14, 15]. In contrast, two other ERF type transcription factors, *SK1* and *SK2*, promote GA accumulation and signaling, triggering internode or petiole elongation, causing shoot emergence [16, 17]. Under relatively shallow submergence, plants predominantly take the low oxygen escape strategy (LOES) [18]. This fast elongation can quickly restore leaf exposure to air, but it might also risk plant death if energy reserves are exhausted before emergence [7]. In addition, because submergence is primarily a low-oxygen stress, morphological changes such as the development of aerenchyma and adventitious roots [19–25], and cellular adjustments that favor low-oxygen detection and energy crisis management [26–28], can all improve plant tolerance to prolonged submergence.

Lotus (*N. nucifera*) is an emergent aquatic plant that is widely cultivated in Asia, for its ornamental flowers, medicinal properties, nutritious tubers, and seeds [29–31]. Lotus can grow in water up to a depth of 2 meters with its leaves emerging well above the surface. For adaptation to aquatic environments, lotus develops aerenchyma structures throughout the plant, which enables internal aeration between shoots and roots [32]. Moreover, the superhydrophobic lotus leaves retain a microlayer of gas under complete submergence [33], referred also as “gas film” or “gas envelope” in rice [34, 35], which enhances CO_2_ supply and favors underwater photosynthesis. Despite these, lotus is still liable to suffer submergence stress during heavy rain seasons, as it is predominantly cultivated in flood-prone low lands and still lake areas [36]. Previous reports have shown that a 12-hours complete submergence of lotus can induce a rapid and robust alteration in the mRNA level of approximately 10% of lotus genes [36]. MicroRNAs (miRNAs) act extensively as regulatory nodes in response to submergence stresses in lotus, modulating the expressions of genes involved in antioxidant systems, hypoxia adaptation, and disease resistance [37]. Nevertheless, little is known about the molecular mechanism of lotus endurance to complete submergence. Moreover, its key responsive strategies remain to be addressed, both genetically and morphologically.

In this study, complete submergence experiments and a time-course transcriptome analyses were performed on two lotus varieties, “China Antique” and “Qiuxing”. “China Antique” is a native lotus variety in China, a typical temperate lotus that flowers in June and July and develops swollen rhizomes in the autumn, whereas “Qiuxing” is a hybrid between tropical and temperate lotus with a longer flowering period from June to October, and its stolons remain unexpanded in autumn. We aimed to investigate lotus tolerance to complete submergence, and to explore its possible strategies to cope with submergence-associated stresses. These results will improve our understanding of the diverse mechanisms of plant responses to submergence and provide valuable genetic sources for the breeding of submergence tolerant lotus cultivars.

## Results

### Endurance capacity of lotus plants under complete submergence

**Figure 1:**
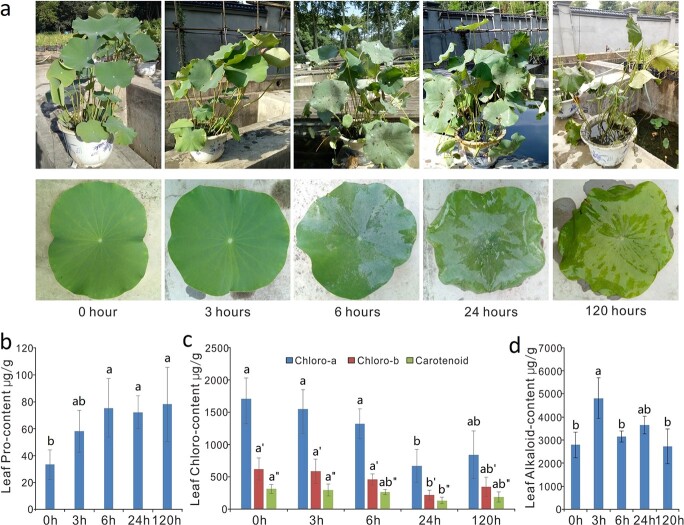
Dynamic changes in “Qiuxing” plants under complete submergence. (a) Phenotypes of submerged lotus plants at 0, 3, 6, and 24 hours and 5 days of submergence. Accumulation of proline (b), chlorophyll (c), and benzylisoquinoline alkaloids (d) in leaves of submerged lotus at different time intervals. Lowercase letters indicate statistically significant differences at *P* < 0.05.

To test the tolerance of lotus plants to complete submergence, flooding experiments were performed on the two lotus cultivars “Qiuxing” and “China Antique”. Three months after potting, plants were completely submerged for up to 15 days. In general, both cultivars had a very similar response to complete submergence. The shapes of plants submerged for three, six, and 24 hours remained similar to those of the non-treated controls; however, their initially water-repellent leaves became gradually wet and greasy. Petioles of newly developed leaves became slim and long, showing an obvious downward bending phenotype at six and 24 hours of submergence (HOS) (Fig. 1a). At five days of submergence (DOS), most lotus leaves became yellowish and soft, and more than half fell off because of their decomposed leaf petioles. At 10 DOS, almost all leaves of submerged plants had fallen off. In addition, submergence also induced cell death in lotus roots and sheathing cataphylls. The color of lotus roots changed gradually from white to brown and black before they died off under prolonged submergence (Fig. S1).

Plants were de-submerged after flooding for different time periods and allowed to recover under routine growth conditions. All plants treated for three hours to five days recovered. Two to three of the five 10-day treated plants died; the remaining plants developed new leaves after two to five days of recovery and survived. However, all plants treated for 12 days failed to develop any new leaves during the two weeks of recovery and subsequently died. The submergence experiment was performed three times, and similar results were observed (Table 1). Thus, the median lethal time of complete submergence for the two lotus varieties was estimated to be approximately 10 days.

**Table 1:** Summary statistics for complete submergence experiments in lotus (*Nelumbo nucifera*)

Duration of complete submergence (days)	Repetition	Number of treated plants	Number of dead plants (var. Qiuxing)	Number of dead plants (var. China Antique)
5	1	5	0	0
	2	5	0	0
	3	5	0	0
10	1	5	3	3
	2	5	3	3
	3	5	2	3
12	1	5	5	5
	2	5	5	5
	3	5	5	5

Proline accumulation is an indicator of various stresses in plants [38]. Accumulation of proline in “Qiuxing” was clearly detected from 3 HOS, before any observable phenotypic changes, and the proline content continuously increased until day five (Fig. 1b). Chlorophyll content is another indicator of plant health and nutritional status, and it decreases in plants under stress conditions [39]. All chlorophyll a, b, and carotenoid contents in lotus leaves were significantly reduced after 24 h of complete submergence (Fig. 1c). In addition, the accumulation of wound-induced alkaloids [30, 40] was significantly increased in the leaves of submerged lotus at 3 HOS, although it later decreased slightly (Fig. 1d).

**Figure 2:**
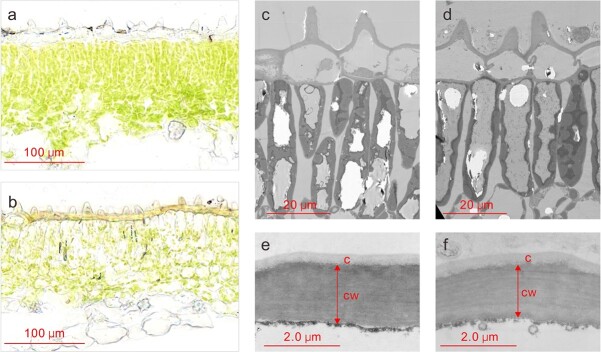
Anatomy of submerged leaves of “Qiuxing” at 48 hours of complete submergence. (a) and (b), Cryo-sections of aerial and aquatic leaves. (c) and (d), TEM images of epidermal and palisade cells of aerial and aquatic leaves. (e) and (f), TEM images showing the adaxial epidermal cell wall (cw) with a cuticle (c) of aerial and aquatic leaves.

Submergence also altered lotus anatomical features. For example, the upper mesophyll cell layers of lotus leaves that developed under water (aquatic leaves) were thinner than those that developed above the surface (aerial leaves), with a much lower chloroplast density (Fig. 2a and b). The epidermal and mesophyll cells of aquatic leaves were slightly smaller, although their shapes remained similar to those of aerial leaves (Fig. 2c and d). In addition, the epidermal cell walls in aquatic leaves were obviously thinner, with an approximately 18% decrease in thickness compared with those in aerial leaves (Fig. 2e and f).

### Time course transcriptome profiles of submerged lotus

To investigate the overall gene expression of lotus plants in response to complete submergence, we constructed 15 RNA-seq libraries for “Qiuxing” at five submergence time intervals: 0 h, 3 h, 6 h, 24 h, and 120 h (5 days). Fully expanded leaves at developmental stage 5 (S5) [30] were sampled (Fig. 1a). On average, 52.46 million single-ended reads were generated from each library, ranging from 46.55 to 60.4 million. Of these single-ended reads, an average of 46.57 million reads, with an average GC content of approximately 47.25%, were mapped to the lotus reference genome [41]. After quality control and filtering, a total of 28 748 unigenes were detected in at least one library. Of these, 3680 genes had not previously been annotated and thus represented novel genes (Table S1, Supplementary data S1). Among these novel genes, 3572 genes were translated with an initial methionine, and they were mostly enriched in the eggNOG terms replication, post translational modification, and signal transduction.

**Figure 3:**
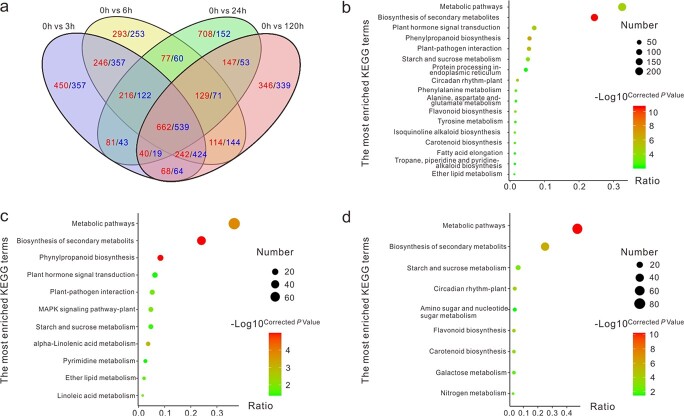
Identification of differentially expressed genes (DEGs) in submerged “Qiuxing” plants. (a) Venn diagrams showing the numbers of DEGs and the shared up- or downregulated DEGs among submergence comparisons. Red and blue colors represent the number of up- and downregulated genes, respectively. (b) Kyoto Encyclopedia of Genes and Genomes (KEGG) enrichment analysis of the 1201 shared DEGs among the four submergence comparisons. (c) and (d), KEGG enrichment analysis of the up- and downregulated DEGs, respectively.

**Figure 4:**
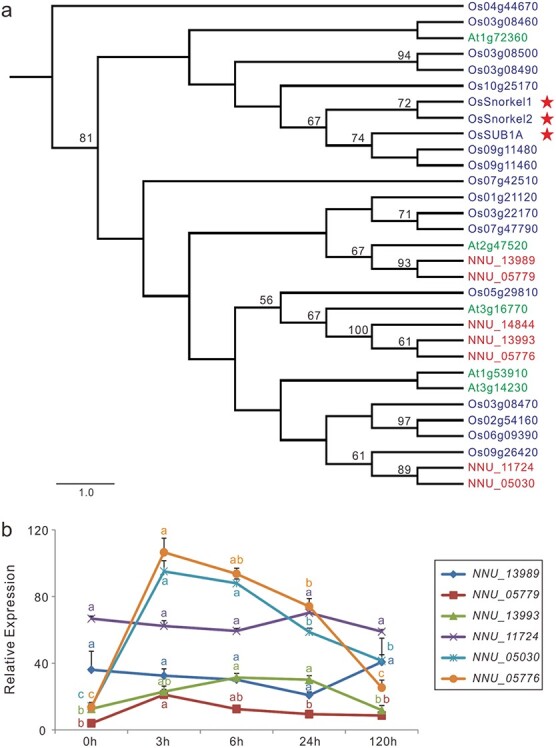
*ERV-VII* genes in lotus. (a) Phylogenetic tree of ERF-VIIs from lotus (red), rice (blue), and *Arabidopsis* (green). Full-length amino acid sequences of ERFs were aligned using MUSCLE, and the phylogenetic tree was constructed with the neighbor-joining method in MEGA7 with 1000 bootstrap replicates. In the phylogenetic tree, only bootstrap values >50% are shown on the branches, and ERFs labeled with red stars are the three rice submergence master regulators. (b) Expression of lotus *ERF-VIIs* in response to complete submergence. Gene expression data were obtained from submerged samples of “Qiuxing” by qRT-PCR, and error bars indicate the standard error (SE) of three biological replicates. Lowercase letters indicate statistically significant differences at *P* < 0.05.

Subsequently, differentially expressed genes (DEGs) were identified between submergence treatments using filter criteria of FDR < 0.01 and fold-change ≥2. A total of 3930 DEGs were identified in the 0 h vs. 3 h comparison, with 2018 and 1912 genes showing up- and downregulation, respectively. Similarly, 3949, 3118, and 3400 DEGs were obtained in the 0 h vs. 6 h, 0 h vs. 24 h, and 0 h vs. 120 h comparisons, respectively. Of these DEGs, 519 were novel genes, and 1201 were commonly up- or downregulated among the four tested comparisons (Fig. 3a). KEGG pathway enrichment analysis showed that these shared DEGs were involved mostly in pathways related to plant metabolism and stress responses (Fig. 3b). “Metabolic pathways”, “biosynthesis of secondary metabolites”, and “plant hormone signal transduction” were the three most significantly enriched pathway terms. Notably, the upregulated DEGs were mostly enriched in the pathways “phenylpropanoid biosynthesis”, “plant hormone signal transduction”, “plant-pathogen interaction”, and “MAPK signaling pathway” (Fig. 3c). Downregulated DEGs were mostly enriched in the terms “starch and sucrose metabolism”, “circadian rhythm”, “amino sugar and nucleotide sugar metabolism”, and “flavonoid biosynthesis” (Fig. 3d).

Gene ontology (GO) term enrichment analysis (*P* ≤ 0.05) was also performed for the 1201 common DEGs. Among the three main GO categories, the commonly upregulated DEGs were mostly enriched in the terms “protein phosphorylation”, “plasma membrane”, “cytosol”, and “protein binding” (Fig. S2a and c). By contrast, the downregulated DEGs were mostly enriched in the terms “response to abscisic acid”, “plasma membrane”, “chloroplast”, and “protein binding” (Fig. S2b and d). Notably, the upregulated DEGs were also enriched in the terms “response to fungus”, “cellular response to hypoxia” and “response to ethylene”, and the downregulated DEGs were enriched in “response to photosynthesis” and “response to water deprivation”.

RNA-seq libraries constructed for the “China Antique” lotus variety also revealed similar gene expression profiles (Table S2, Supplementary data S2). Among the four tested comparisons, 2443 DEGs were commonly up- or downregulated (Fig. S3a). These DEGs were mostly enriched in the pathways “metabolics”, “biosynthesis of secondary metabolites”, and “carbon metabolism” (Fig. S3b). Notably, DEGs identified in the two varieties were enriched predominantly in similar or closely related KEGG terms. This phenomenon became more evident when the up- and downregulated DEGs were evaluated separately. Seven of the ten enriched KEGG terms for the upregulated DEGs and seven of the eight terms for the downregulated DEGs in “Qiuxing” were shared with those in “China Antique” (Fig. S3c and d). Most key genes involved in the submergence response in “China Antique” also showed similar expression patterns in “Qiuxing” at the five submergence time points (Fig. S4, S5). Because of the high transcriptome similarity between these two varieties, we have presented the data obtained from “Qiuxing” in subsequent sections of this work.

### Identification of lotus *ERF-VII* genes

Given the importance of ERF transcription factors in plant adaptation to flooding and hypoxia [13, 16], we initially searched for *ERF* family genes in lotus using BLAST with the *Arabidopsis* AP2/ERF domain (59 amino acids) as a query sequence. A total of 125 *ERF* genes were identified in the lotus genome. These *ERFs* were phylogenetically classified in 10 groups together with representative rice genes from each ERF group [42] (Fig. S6). Seven NnERFs clustered together with OsSUB1A and an OsERF-VII and were therefore deemed to be lotus ERF-VIIs. Subsequently, a fine neighbor-joining phylogenetic tree was constructed using only the ERF-VII protein sequences (Fig. 4a), including 15 rice ERF-VIIs (*O. sativa* ssp. *japonica*), 5 *Arabidopsis* ERF-VIIs, 7 lotus ERF-VIIs, 1 OsSUB1A, and 2 OsSnorkels (*O. sativa* ssp. *indica*). One rice ERF (Os04g44670) from group I was used as the outgroup. The ERF-VII proteins were clustered into two large groups. All three rice submergence master regulators, OsSUB1A and two OsSnorkels, were classified into one large group, together with *Arabidopsis* HRE1 (HYPOXIA RESPONSIVE ERF protein At1g72360) and six rice ERF-VIIs. All seven lotus ERV-VIIs were classified into the other large group.

**Figure 5:**
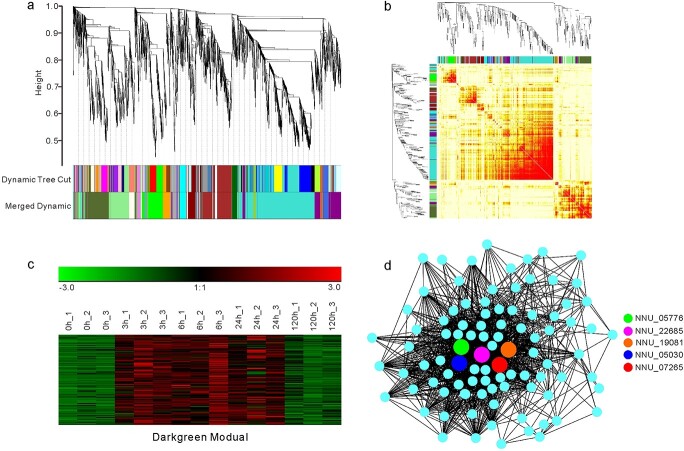
Weighted gene co-expression network analysis (WGCNA) for DEGs identified in “Qiuxing” lotus under complete submergence. (a) Identification of co-expression modules using a hierarchical clustering tree. The 21 identified modules are indicated with different colors. (b) Heatmap showing the topological overlap matrix of 1500 randomly selected DEGs. The faded color indicates lower overlap, and red indicates higher overlap. (c) Expression patterns of DEGs in the “Darkgreen” co-expression module. (d) Topological illustration of the “Darkgreen” co-expression module. Colored nodes represent the five hub genes associated with plant responses to biotic and/or abiotic stresses.

Of the seven *NnERF-VIIs*, *NNU_14844* was not expressed in lotus leaves. *NNU_11724* and *NNU_13989* were constitutively expressed in lotus leaves, showing little regulation under submergence. By contrast, four *NnERF-VIIs*, including *NNU_05776*, *NNU_05030*, *NNU_13993*, and *NNU_05779*, were significantly induced by submergence (Fig. 4b). These four *NnERF-VIIs* could be highly relevant for lotus adaptation to complete submergence.

**Figure 6:**
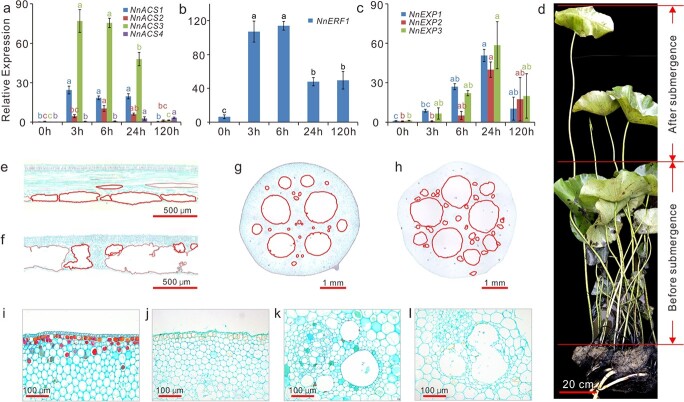
Submergence significantly enhances lotus petiole elongation and aerenchyma formation. Genes encoding ACC synthases (NnACSs) (a), ethylene marker protein NnERF1 (b), and expansins (NnEXPs) (c) were significantly upregulated. (d) Submerged lotus plants with dramatically elongated petioles. (e–l), Safranin O/fast green staining of cross sections of lotus leaves and petioles developed under routine conditions (e, g, i, k), or complete submergence (f, h, j, l). Aerenchyma in leaf and petiole sections are marked with red circles (e–h). Enhanced resolution images of petiole cortex cells (i and j) and aerenchyma (k and l). Gene expression data were obtained from submerged samples of “Qiuxing” by qRT-PCR, and error bars indicate the SE of three biological replicates. Lowercase letters indicate statistically significant differences at *P* < 0.05.

### Mining of submergence-responsive genes by WGCNA analysis

To explore lotus genes involved in the response to complete submergence, weighted gene co-expression network analysis (WGCNA) was performed for all DEGs identified in the “Qiuxing” variety. Based on the DEG hierarchical clustering and the cluster heat map, 21 gene co-expression modules were identified among the 6273 DEGs (Fig. 5a and b). DEGs located in the same module appeared to have very similar expression patterns and were therefore deemed to be tightly co-regulated. For example, the “Brown” module (containing 707 DEGs) represented DEGs that were significantly upregulated at 3 HOS and comprised mainly genes involved in processes of the endoplasmic reticulum lumen and response to endoplasmic reticulum stress (Fig. S7a and e). Similarly, the “Darkgreen” (114 DEGs) and “Orange” (200 DEGs) modules were dramatically enhanced at 3–24 HOS and 5 DOS, respectively (Fig. S7b and c). Moreover, the “Turquoise” module (1832 DEGs) represented DEGs that were dramatically repressed by complete submergence at all time intervals. These DEGs were mostly involved in response to abiotic stimulus and cellular processes (Fig. S7d).

DEGs in the “Darkgreen” module were consistently upregulated from 3 to 24 HOS (Fig. 5c) and were therefore deemed to be tightly associated with the lotus submergence response. Of the 114 DEGs in the “Darkgreen” module, seven encoded transcription factors, including three ethylene-responsive factors (ERFs), two bHLHs, one WRKY, and one HBI (Supplementary Data S3). This module also contained six genes that are known to be involved in submergence responses and pathogen resistance, including one GA20 oxidase (*NNU_26591*), two LRR receptor-like serine threonine kinases (*NNU_11053*, *NNU_04059*), one pathogen-related protein-like protein (*NNU_04043*), one disease resistance RPP13-like protein (*Nelumbo_nucifera_newGene_9967*), and one zinc-finger homeodomain protein (*NNU_07265*).

A total of 95 DEGs in the “Darkgreen” module had WGCNA edge weights over 0.10. Among these DEGs, 1393 pairs of co-expression edges were linked (Fig. 5d). The top five most connected genes (hub genes) were *NNU_19081*, *NNU_22685*, *NNU_07265*, *NNU_05776* and *NNU_05030*, with 79, 72, 72, 68 and 55 connections, respectively. These five genes are widely involved in plant response to biotic and abiotic stresses and may be highly associated with the lotus response to complete submergence. Notably, *NNU_05776* and *NNU_05030* encode ERF-VII proteins, a class of ethylene response factors that regulate a large set of genes involved in flooding and low oxygen response [12, 16]. *NNU_19081* is a receptor-like protein kinase (RLK) that resembles BR1 in *Arabidopsis* and is potentially involved in brassinosteroid-mediated stress responses [43]. *NNU_22685* encodes a peroxidase 17-like protein that is potentially involved in carbohydrate transport and metabolism. *NNU_07265* encodes a zinc-finger homeodomain protein 9 (ZF-HD9), and overexpression of its *Arabidopsis* homolog can increase tolerance to salt and drought stresses [44].

**Figure 7:**
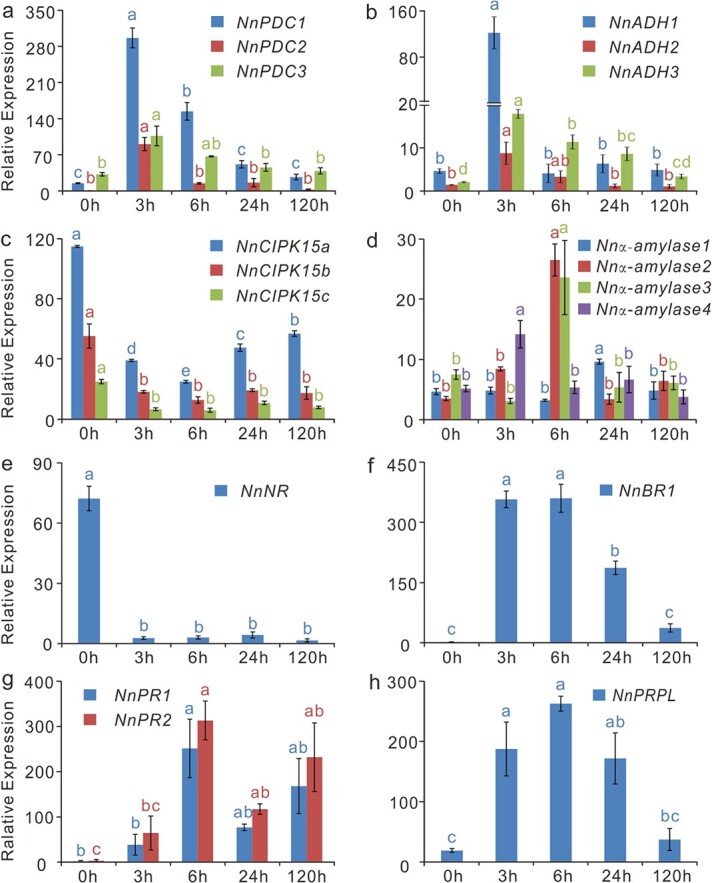
Submergence altered anaerobic fermentation and pathogen resistance in lotus. (a–e) Expression of lotus genes encoding pyruvate decarboxylases (PDCs), alcohol dehydrogenases (ADHs), calcineurin B-like protein-interacting protein kinase 15s (CIPK15s), α-amylases, and a nitrate reductase (NR). (f–h) Expression of lotus genes encoding an NnBR1, two PATHOGENESIS-RELATED GENE1s (NnPR1s), and a pathogen-related protein-like protein (NnPRPL). Gene expression was obtained from submerged samples of “Qiuxing” by qRT-PCR, and error bars indicate the SE of three biological replicates. Lowercase letters indicate statistically significant differences at *P* < 0.05.

### Submergence significantly enhances lotus petiole elongation and aerenchyma formation

To investigate possible response strategies of lotus to complete submergence, we first screened for the expression of submergence genes associated with the “escape” strategy. ACC synthases (ACSs) catalyze a rate limiting step in plant ethylene biosynthesis [45, 46]. The lotus genome contains four ACSs (*NNU_25645*, *NNU_12026*, *NNU_13826*, and *NNU_16817*), all of which were significantly induced by complete submergence, with an approximately 1000-fold increase at 3 HOS (Fig. 6a). This high expression was sustained until 24 HOS and then decreased to approximately basal levels at 5 DOS. Consistent with this result, the expression of the ethylene marker gene *NnERF1* (*NNU_10421*) [47, 48] was also significantly upregulated and remained at high levels until 5 DOS (Fig. 6b), indicating a continuous accumulation of the gaseous phytohormone ethylene during complete submergence. In addition, three genes encoding expansins (*EXPs*), *NNU_07293*, *NNU_04640*, and *NNU_23652*, were markedly upregulated (Fig. 6c). Intriguingly, accumulation of *EXP* mRNA has been reported to be strongly correlated with submergence-induced elongation [49, 50].

Petioles of submerged plants rapidly elongated on the first and second day of submergence treatment, consistent with internal gene expression levels. New leaves that developed under complete submergence were clearly taller, and their petioles were approximately 20 cm longer than those which developed under normoxic conditions. Notably, three of the submerged “Qiuxing” and five of the submerged “China Antique” plants extended their leaves above the water surface. The petioles of these plants had elongated dramatically under submergence to about 40–100 cm longer than those of older leaves (Fig. 6d). These observations clearly suggest that lotus plants used the “escape” strategy during complete submergence.

As a wetland species, lotus routinely forms aerenchyma in the leaves and petioles (Fig. 6e and g), providing a system of interconnected channels from leaves to roots. Leaves and petioles that developed under complete submergence had markedly more extensive aerenchyma (Fig. 6f and h), with some aerenchyma spaces clearly forming through connections of neighboring air holes (Fig. 6f, h, and l). In addition, the epidermal cells of lotus petioles that developed under submergence conditions were almost degenerated compared with those of petioles that developed in air (Fig. 6i and j).

### Submergence alters lotus anaerobic fermentation and pathogen resistance

We next investigated the expression of pathway genes relevant to metabolic adaptation to complete submergence. Under complete submergence, oxygen availability is limited, and plants must use the less efficient anaerobic respiration pathway (glycolysis and fermentation) to maintain a necessary energy supply. Pyruvate decarboxylase (PDC) and alcohol dehydrogenase (ADH) catalyze two key steps in the process of fermentation, converting pyruvate produced during glycolysis into acetaldehyde and ethanol, respectively [4, 26]. During complete submergence, lotus exhibited strong expression of genes encoding NnPDCs (*NNU_12640*, *NNU_13080*, and *NNU_00240*) and NnADHs (*NNU_14241*, *NNU_14240*, and *NNU_16725*) at 3 HOS, suggesting a sharp and strong induction of glycolysis and fermentation (Fig. 7a and b). However, the expression of these genes decreased under prolonged submergence. Dramatic downregulation of expression was also observed in genes encoding calcineurin B-like protein-interacting protein kinase15s (NnCIPK15s; *NNU_19262*, *NNU_00930*, and *NNU_19321*), which regulate carbohydrate breakdown (Fig. 7c). In addition, four genes encoding lotus alpha-amylases (*NNU_23926*, *NNU_07806*, *NNU_07200*, and *NNU_13572*) were initially induced at 6 HOS but subsequently suppressed after prolonged submergence (Fig. 7d). Interestingly, the expression of lotus nitrate reductase (NnNR, *NNU_07777*), another central homeostatic regulator of hypoxic stresses and a reverse regulator of ERF VII proteins [51], was sharply downregulated (Fig. 7e). This signaled the detection of hypoxic stress by lotus and an attempt to eliminate excess NO to regulate anaerobic metabolism. Thus, our digital gene expression data indicate that lotus glycolysis and fermentation were almost immediately activated at the onset of complete submergence to supply energy for petiole elongation. However, this anaerobic respiration seemed to be maintained at relatively low levels during later submergence stages.

Similarly, genes that modulate pathogen resistance and ROS scavenging were also adjusted in submerged lotus. At first, the expression of the WGCNA hub gene *NnBR1* (*NNU_19081*), which is involved in pathogen resistance, was dramatically elevated by complete submergence (Fig. 7f). Likewise, over 100-fold upregulation was observed in the expression of lotus *PATHOGENESIS-RELATED GENE 1* genes (*NnPR1s, NNU_24605*, and *NNU_24602*), molecular markers for systemic acquired resistance (SAR) [52] in submerged lotus (Fig. 7g). Furthermore, significant upregulation was also observed in the expression of a pathogen-related protein-like gene (*NnPRPL, NNU_04043*) (Fig. 7h) and three peroxidases (*NnPODs*, *NNU_18800*, *NNU_12989*, and *NNU_04048*; Fig. S8). Taken together, these results suggested that complete submergence could increase the expression of lotus resistance genes and ROS-elimination genes to facilitate submergence adaptation.

### The lotus response to submergence is modulated by phytohormones

Regulation of plant response to submergence has been tightly linked to three phytohormones: ethylene, abscisic acid (ABA), and gibberellic acid (GA) [53, 54]. To estimate levels of these three hormones under complete submergence, the expression of their key biosynthetic genes was investigated.

**Figure 8:**
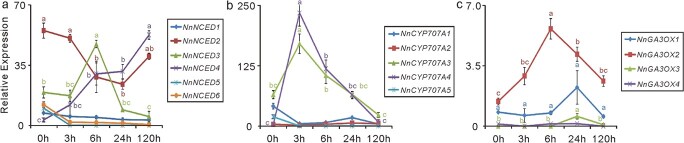
Lotus submergence responses are also modulated by ABA and GA. qRT-PCR was used to measure expression levels of rate-limiting genes involved in the biosynthesis of ABA (a, b) and GA (c). Samples were taken from “Qiuxing”, and the error bars indicate the SE of three biological replicates. Lowercase letters indicate statistically significant differences at *P* < 0.05.

All four putative *NnACSs* involved in ethylene biosynthesis were significantly induced by complete submergence (Fig. 6a). *NnACS3* is the key member of the *NnACS* genes, and its expression was highest between 3 and 24 HOS. NCED is a rate-limiting enzyme in ABA biosynthesis [55], and it is putatively encoded by a series of six genes in lotus, designated *NnNCED1*–*NnNCED6* (*NNU_03160*, *NNU_03163*, *NNU_23070*, *NNU_23071*, *NNU_02734*, and *NNU_11066*). Among them, *NnNCED2*, *NnNCED3*, and *NnNCED4* are expressed mainly in lotus leaves. Under complete submergence, *NnNCED2* was significantly downregulated from 3 to 24 HOS, whereas *NnNCED4* was significantly upregulated at 5 DOS. The expression levels of *NnNCED3* fluctuated, with an expression peak at 6 HOS (Fig. 8a). In contrast to the expression of ABA biosynthetic genes, the expression levels of two major ABA degradation genes, *NnCYP707A*s (*NNU_23085* and *NNU_02459*), were very similar: they were rapidly induced at 3 HOS, then sharply decreased at all later time intervals (Fig. 8b). This result suggested that ABA concentration in submerged lotus was likely to decrease at the beginning of the submergence treatment. Furthermore, the expression of four GA rate-limiting biosynthetic genes, designated *NnGA3OX1–NnGA3OX4*, was relatively low in lotus leaves. These four genes were initially upregulated by complete submergence between 6 and 24 HOS but were later downregulated after prolonged submergence (Fig. 8c). Taken together, our data suggest a sharp increase in ethylene accumulation, a slight increase in GA content, and a slight decrease in ABA content in the early stages of submergence.

**Figure 9:**
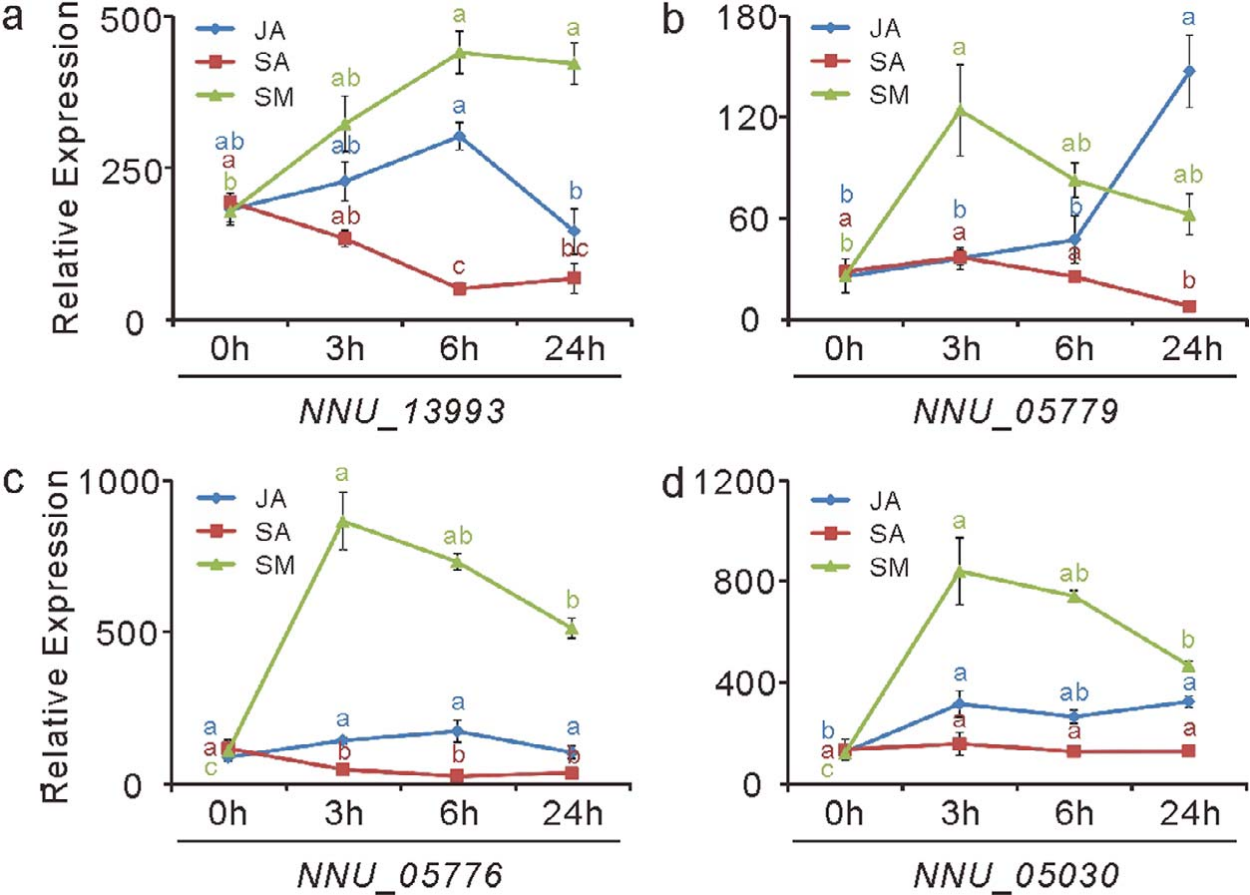
Expression of lotus *ERF-VII* genes (*NNU_13993*, *NNU_05779*, *NNU_05776*, and *NNU_05030*) in response to JA, SA, and submergence treatment. Samples were taken from “Qiuxing”, and the error bars indicate the SE of three biological replicates. Lowercase letters indicate statistically significant differences at *P* < 0.05.

We also examined the expression of the four lotus submergence-responsive *NnERF-VII*s upon SA, JA, and submergence treatments using our previously reported data [56]. Of the four genes, *NNU_05030* and *NNU_05779* were dramatically upregulated by complete submergence treatment. However, these genes were less extensively regulated by SA and JA treatment (Fig. 9, a–d). JA treatment had no obvious effect on *NNU_05776* and *NNU_13993* expression but enhanced *NNU_05779* and *NNU_05030* expression. By contrast, SA treatment slightly downregulated the expression of *NNU_13993*, *NNU_05779*, and *NNU_05776* but had no effect on *NNU_05030* expression. These results suggest that, in addition to the gaseous phytohormone ethylene, JA may also be involved in the regulation of lotus responses to complete submergence.

## Discussion

Lotus is an emergent aquatic plant that grows in the margins of still lakes, ponds, and slow-moving rivers. In China, lotus is predominantly cultivated in wetlands and flood-prone areas along the Yangtze River, where it is predisposed to complete submergence during rainy seasons, resulting in significant production loss [36]. Our study revealed that the “Qiuxing” and “China Antique” lotus varieties had an overall low tolerance to complete submergence, with an average lethal time of about 10 days, shorter than the 40–100 day lethal times observed in other wetland species [57] but comparable to the 7–16 day lethal time reported in rice [12]. A previous study reported a wide variation in the median lethal times observed under submergence + dark conditions (4–11.2 days) and those observed under submergence + light conditions (16–20 days) among different *Arabidopsis* accessions [58]. Thus, a higher capacity for enduring submergence is likely to occur in deep-water varieties of other plant species such as lotus.

Our complete submergence experiment showed that the aerial organs of lotus died primarily because of petiole collapse, which began at approximately 3 DOS. In the meantime, extensive cell death occurred in the underground organs. De-submerged lotus plants with both dead aerial and underground organs did not survive. However, a few plants that were de-submerged after prolonged submergence ultimately developed leaves and survived.

The transcriptome profiles of submerged lotus leaves revealed a total of 28 748 unigenes. Of these, 6735 genes (DEGs) exhibited dramatic changes in gene expression during submergence treatment, indicating a significant effect of submergence on lotus gene expression. KEGG analysis showed that the top 6 most enriched pathways were “metabolic pathways”, “biosynthesis of secondary metabolites”, “plant hormone signal transduction”, “phenylpropanoid biosynthesis”, “plant-pathogen interaction”, and “starch and sucrose metabolism”. This suggests that signal transduction, regulation of energy supply and consumption, and perception of pathogen infection are the main coping strategies of lotus plants under complete submergence.

Our integrated morphological analysis and time course transcriptome profiling of key submergence-responsive genes revealed pivotal response strategies of lotus to submergence stress. First, an “escape” strategy was evidently employed. Under complete submergence, petioles of newly emerged leaves elongated rapidly between 1 and 3 DOS, allowing lotus leaves to regain contact with oxygen-rich air. Emerged leaves, acting as snorkels, facilitated the entrance of oxygen and the release of gases such as ethylene and ethane. In this study, leaves of nearly one fifth of the treated lotus plants extended above the water surface at 3 DOS. These plants eventually survived and grew normally after de-submergence. Rapid petiole elongation has also been observed in deep-water rice and *Rumex palustris* [16, 57], activated by low oxygen concentration and promoted by ethylene and GA phytohormones. Ethylene-responsive ERF-VII transcription factors, including the SNORKELs, act as key regulators of escape responses [16]. The lotus genome encodes 7 ERF-VIIs, four of which were significantly elevated by submergence. Consistent with this result, four ethylene biosynthetic genes (*NnACS1–4*) and three lotus expansin genes (*NnEXP1–NnEXP3*) were highly induced, indicating a burst of ethylene production and the elongation of lotus petioles under complete submergence [48, 59].

It is curious that none of the lotus ERF-VIIs were phylogenetically grouped with rice SUB1A or Snorkels, although ERF subfamily members had clearly evolved prior to the speciation of rice, *Arabidopsis*, and lotus [60] (Fig. S6, Fig. 4). However, we cannot exclude the possibility that these NnERFs were involved in the lotus response to submergence, based on previous studies in *Arabidopsis*. The *Arabidopsis* genome encodes five *ERV-VII* genes. Although only HRE1 (At1g72360) was located in the same group as rice SUB1A and Snorkels, all *Arabidopsis* ERF-VIIs have been demonstrated to participate in adaptation to submergence according to studies on overexpression lines and loss-of-function mutants [61].

Similar to *R. palustris* [62], submerged lotus displayed morphological, anatomical, and biochemical changes. Lotus plants under complete submergence developed thinner specialized leaves and slimmer petioles with a higher density of aerenchyma compared with those that developed above the water surface. The formation of aerenchyma normally occurs simultaneously with leaf/petiole elongation, and it is stimulated by submergence-induced ethylene [22]. In addition, cell walls in aquatic leaves were approximately 20% thinner. These anatomical and morphological traits increased underwater photosynthesis and internal aeration [32], thus facilitating plant endurance of complete submergence. Complete submergence also rapidly induced the expression of two fermentation genes from the *NnPDCs* and *NnADHs* at 3 HOS. Their expression was subsequently downregulated and maintained at a lower level at later submergence time points. Similarly, the expression levels of four lotus *alpha-amylases* were initially elevated and later reduced and maintained at lower levels. These results suggest that lotus glycolysis and fermentation were quickly activated at the early stages of submergence to supply energy for petiole elongation. However, anaerobic respiration was maintained at a low level during later stages of submergence to conserve carbohydrate supply and increase submergence endurance.

In the “escape” syndrome, plant petioles or internodes elongate rapidly, facilitating plant survival by restoring leaves to the oxygen-rich air above the water surface. However, this process comes at a high energy cost, especially when plants are under complete submergence and must use inefficient anaerobic respiration [3, 7]. It is likely that the rapid elongation of lotus petioles during early submergence led to high energy consumption, resulting in plant death when leaves failed to emerge out of the water. This could explain why lotus, despite being an aquatic plant, has a relatively short lethal time compared with many other wetland species under complete submergence.

Increased innate immunity was also observed in submerged lotus. For example, a large set of DEGs were enriched in the plant-pathogen interaction pathway. Among these enriched DEGs, the expression levels of two lotus SAR marker genes (*NnPR1* and *NnPR2*) and two pathogen-related genes (*NnBR1* and *NnPRPL*) were dramatically elevated over 100-fold under submergence. This is consistent with a previous report showing enhanced resistance to rot disease in lotus seedlings pretreated by submergence [36]. Consistent with this study, we also observed a burst of lotus POD gene expression, indicating an enhancement of the ability of lotus plants to eliminate excessive ROS produced under submergence.

Furthermore, plant underwater elongation is known to be mediated by the production and signaling of GA [17]. Complete submergence induces the biosynthesis of ethylene, which in turn downregulates ABA production and stimulates GA production [53]. Lotus gene expression patterns implied a burst in ethylene production, a slight increase in GA, and a decrease in ABA during the early submergence period between 3 and 24 HOS. This was consistent with the observed petiole elongation phenotypes. In addition, we observed a slight upregulation in the expression of two submergence-responsive *NnERFs* upon JA treatment, indicating a possible role for JA in the mediation of lotus submergence responses.

In summary, our study shows that lotus plants have a low tolerance to complete submergence, with a median lethal time of approximately 10 days. Time-course transcriptomic analysis identified a number of key genes that are potentially involved in the lotus submergence response. The data presented in this research reveal that the low oxygen “escape” strategy was predominantly adopted by lotus to cope with complete submergence–induced stresses. It remains to be determined, however, whether a large variation in tolerance exists in a wider collection of natural lotus germplasm and whether lotus employs different acclimation strategies at different depths of complete submergence.

## Materials and methods

### Plant materials

The two lotus varieties “China Antique” and “Qiuxing” were used in this study. Rhizomes were harvested in March, potted in 40 cm × 50 cm (diameter × depth) containers in April, and grown under conditions of natural light and temperature at the Wuhan Botanical Garden of the Chinese Academy of Sciences (Wuhan, Hubei Province, China). Growth pots were all set outdoors on flat ground, filled with 30 cm of soil, and covered with water to a 20-cm depth. Seedlings were routinely supplied with water and fertilizer.

### Submergence treatments

Complete submergence treatments were performed on a sunny day in outdoor concrete pools (1.5 m wide × 2.5 m long × 2 m deep) under a natural day-night rhythm. Three months after potting, plants of “China Antique” and “Qiuxing” were completely submerged at 10 am for a period of 3 h, 6 h, 1 day, 2 days, 5 days, 10 days, 12 days, or 15 days, with the top leaves maintained about 40 cm below the water surface. Forty lotus plants of each variety were treated. At each time point, five plants were de-submerged and set in a separate pool to recover under normal growth conditions for two weeks. As a control, five plants were grown in a separate pool with normal water levels. For RNA sequencing, laminas at developmental stage 5 (S5) [30] were sampled from completely submerged plants at different time points (10 am [0 h], 11 am [1 h], 1 pm [3 h], and 4 pm [6 h] on the treatment day, 10 am [24 h] on the following day, and 10 am on the 5th day [5 days]) and immediately frozen in liquid nitrogen, then stored at −80°C until use. For anatomical observations, both petioles and laminas showing significantly elongated phenotypes were sampled at 48 h of submergence.

### Measurement of photosynthetic rate and chlorophyll, proline, and alkaloid contents

Leaf chlorophyll, proline, and alkaloid contents were measured in samples after submergence. Total leaf chlorophyll was extracted with 80% acetone, and the contents of chlorophyll a and b were determined by measuring the absorbance at 646.6 nm and 663.6 nm with a spectrophotometer as described previously [63]. The contents of alkaloids and free proline were determined as described previously [30, 64]. For each time point, at least 3 biological replicates were assayed, each with 3 technical replicates.

### Phenotypic analysis of lotus leaves and petioles

The anatomy of lotus leaves and petioles was observed with a light microscope (Leica DMi8, USA) and a transmission electron microscope (TEM; Hitachi HT7800, Japan). Lotus leaf and petiole samples at developmental stage 5 (S5) were taken from plants grown under routine conditions and after 48 h of complete submergence. For frozen tissue specimens, fresh samples were embedded with Tissue-Tek (Sakura Finetek, CA, USA), immediately frozen at −20°C, and sliced into 20-μm sections with a Cryostar NX50 cryostat (Thermo, CA, USA). For safranin O/fast green staining, fresh samples were fixed in FAA solution for 2 days, dehydrated with ethanol, and embedded in paraffin. Sections of 10 μm thickness were cut, stained with 1% safranin O for 10 min, and then counterstained with 0.1% fast green solution for 5 min. For TEM observation, samples of approximately 1 mm [3] were fixed with 1% OsO_4_ in 0.1 M phosphate buffer (pH 7.4) for 7 h at room temperature, dehydrated with ethanol, embedded in EMBed 812 (SPI Chem, PA, USA), sliced into 60–80-nm sections, and stained with a 2% uranium acetate saturated alcohol solution in the dark for 8 min.

### RNA extraction, RNA-seq library construction, and sequencing

Fifteen RNA sequencing libraries were constructed from submerged lotus leaves collected after 0 h, 3 h, 6 h, 24 h, and 120 h. Three biological replicates were used for each time point. Approximately 100 mg of frozen lotus leaf tissue was homogenized in liquid nitrogen with a mortar and pestle. Total RNA was isolated with the Tiangen RNA Extraction Kit with the RNase-Free DNase Set (Tiangen Biotech, Beijing, China). RNA integrity was confirmed by 1% agarose gel electrophoresis. RNA purity and concentration were evaluated using a NanoDrop Lite spectrophotometer (Thermo Scientific, CA, USA) and a Bioanalyzer 2100 system (Agilent Technologies, Santa Clara, CA, USA). Library construction and RNA sequencing were performed using sequencing by synthesis technology based on the Illumina next-generation sequencing platform (Biomarker Technologies Corporation, Beijing, China). All raw data generated in this research have been submitted to the Sequence Read Archive under BioProject ID PRJNA723672.

### RNA-seq data analysis

After filtering adapter sequences and low-quality regions, clean reads were mapped to the reference genome of sacred lotus [41] using HISAT2 [65] and assembled with StringTie [66]. Gene expression levels were evaluated with Fragments Per Kilobase of transcript per Million fragments mapped (FPKM) [67]. DEGs were identified using the DEGseq R package [68] with filtering criteria of false discovery rate (FDR) < 0.01 and fold-change ≥2. Gene Ontology (GO) and Kyoto Encyclopedia of Genes and Genomes (KEGG) pathway enrichment analyses were performed with cutoffs of *P* < 0.01 and *P* < 0.05 for significant biological processes and pathways, respectively. Expression data for all genes at all submergence time points are provided in supplementary data S4 and S5 for “Qiuxing” and “China Antique”, respectively.

Weighted gene co-expression network analysis (WGCNA) was performed using programs in the BMKCloud platform (www.biocloud.net). All DEGs identified in this study were included in the WGCNA analysis. The parameters for WGCNA were set as follows: Power = 9; Hierarchical clustering tree, Dynamic Hybrid Tree Cut algorithm; Minimum module size, 30; and Minimum height for merging modules, 0.25. The gene expression patterns were normalized and visualized using Genesis software [69], and the GO enrichment analysis of DEGs in co-expression modules was performed using the KOBAS 3.0 database [70]. The co-expression networks were visualized with Cytoscape software 3.4.0 [71].

### Quantitative real-time PCR

Total RNA extraction and cDNA synthesis were performed with the RNAprep Pure Plant Kit (Tiangen Biotech, Beijing, China) and Transcript One-Step gDNA Removal and cDNA Synthesis SuperMix (TransGen Biotech, Beijing, China), respectively, according to the manufacturers’ instructions. Quantitative real-time PCR (qRT-PCR) was performed using the Applied Biosystems StepOnePlus Real-Time PCR System (Thermo Fisher Scientific, Waltham, MA USA) and the TB Green Premix Ex Taq II enzyme mix (TaKaRa, Dalian, China). The amplification program was set as previously described, and the *NnActin2* gene was used as a constitutive control [40]. Relative expression of genes was calculated with the 2^−∆∆Ct^ method [72], and all analyses were performed with three biological replicates. The primers used in the study are listed in Table S3.

## Acknowledgments

This project was supported by funds from the National Natural Science Foundation of China (Grant Nos. 31772353, 31700262, and 32070336), the Key Research Program of Frontier Science Chinese Academy of Science (Grant No. QYZDB-SSW-SMC017), and the Youth Innovation Promotion Association CAS (Grant No. 2017390). We thank BMKCloud (http://www.biocloud.net) for providing a platform for data analysis.

## Author contributions

M.Y., X.D. and D.Y. conceived and designed the experiments. X.D., D.Y., H.S., H.Y.S., Y.X., Y.W., J.M., M.Z. and J.L. performed the experiments. X.D. wrote the paper. J.L., M.Y. and Y.L.L. revised the manuscript.

## Data availability

Supplementary information accompanies the manuscript can be found on the *Horticulture Research* website.

## Conflict of interest statement

The authors declare no competing financial interests.

## Supplementary data

Supplementary data is available at *Horticulture Research* online.

## Supplementary Material

Web_Material_uhac001
