# Supplementary material for: Time-course analysis and transcriptomic identification of key response strategies of Nelumbo nucifera to complete submergence
Source: Hortic Res. 2022 Feb 11;9:uhac001. doi: 10.1093/hr/uhac001 (PMC8973275; doi:10.1093/hr/uhac001)
Supplement: Web_Material_uhac001 [file web_material_uhac001.zip › Supplementary tables and figures.docx]

**Table S1.** Summary of RNA sequence data obtained from ‘Qiuxing’ leaves under submergence treatments.

| Sample | No. of reads (million) | | No. of mapped clean reads (million) | | GC content of clean reads | No. of transcripts | |
| --- | --- | --- | --- | --- | --- | --- | --- |
|  | Total | Mapped | Single-mapped | Multiple-mapped |  | Total | Novel (Percentage) |
| SY0h_1 | 50.60 | 46.33 | 45.10 | 1.24 | 47.42% | 22407 | 2586 (11.5%) |
| SY0h_2 | 56.09 | 50.13 | 49.12 | 1.01 | 47.58% | 22793 | 2719 (9.7%) |
| SY0h_3 | 56.95 | 52.13 | 50.95 | 1.18 | 47.19% | 22673 | 2737 (12.1%) |
| SY3h_1 | 52.83 | 47.95 | 41.20 | 0.75 | 47.34% | 22832 | 2771 (12.1%) |
| SY3h_2 | 49.14 | 44.66 | 44.03 | 0.64 | 46.89% | 22874 | 2801 (12.2%) |
| SY3h_3 | 52.83 | 48.07 | 47.37 | 0.71 | 46.67% | 22498 | 2707 (12.0%) |
| SY6h_1 | 51.14 | 45.81 | 45.06 | 0.75 | 46.97% | 22598 | 2711 (12.0%) |
| SY6h_2 | 49.57 | 44.99 | 44.24 | 0.76 | 46.57% | 22301 | 2700 (12.1%) |
| SY6h_3 | 46.78 | 42.27 | 41.65 | 0.62 | 46.93% | 23140 | 2791 (12.1%) |
| SY24h_1 | 46.78 | 42.17 | 41.51 | 0.66 | 47.13% | 23296 | 2806 (12.0%) |
| SY24h_2 | 46.55 | 41.26 | 40.57 | 0.67 | 47.12% | 22256 | 2688 (12.1%) |
| SY24h_3 | 59.35 | 53.58 | 52.67 | 0.91 | 47.47% | 23379 | 2825 (12.1%) |
| SY120h_1 | 60.40 | 51.44 | 50.50 | 0.93 | 47.68% | 23278 | 2793 (12.0%) |
| SY120h_2 | 56.12 | 43.06 | 42.31 | 0.74 | 48.56% | 21741 | 2449 (11.3%) |
| SY120h_3 | 51.79 | 44.63 | 43.88 | 0.75 | 47.23% | 22127 | 2645 (12.0%) |

**Table S2.** Summary of RNA sequence data obtained from ‘China Antique’ leaves under submergence treatments.

| Sample | No. of reads (million) | | No. of mapped clean reads (million) | | GC content of clean reads | No. of transcripts | |
| --- | --- | --- | --- | --- | --- | --- | --- |
|  | Total | Mapped | Single-mapped | Multiple-mapped |  | Total | Novel (Percentage) |
| G0h_1 | 41.77 | 40.59 | 38.85 | 1.74 | 47.19% | 20743 | 2100 (10.1%) |
| G0h_2 | 42.61 | 41.08 | 39.37 | 1.71 | 46.96% | 21445 | 2267 (10.6%) |
| G0h_3 | 44.44 | 43.04 | 41.31 | 1.73 | 47.26% | 21167 | 2206 (10.4%) |
| G3h_1 | 40.27 | 38.86 | 37.71 | 1.15 | 46.25% | 21093 | 2273 (10.8%) |
| G3h_2 | 45.76 | 43.58 | 42.15 | 1.43 | 46.92% | 22052 | 2261 (10.3%) |
| G3h_3 | 47.36 | 45.31 | 44.34 | 0.97 | 46.55% | 22136 | 2311 (10.4%) |
| G6h_1 | 40.13 | 37.96 | 36.70 | 1.26 | 46.73% | 21029 | 2243 (10.7%) |
| G6h_2 | 42.97 | 41.17 | 39.64 | 1.53 | 46.36% | 21292 | 2310 (10.8%) |
| G6h_3 | 41.61 | 39.65 | 38.56 | 1.09 | 46.48% | 21465 | 2301 (10.7%) |
| G24h_1 | 49.57 | 47.85 | 46.35 | 1.49 | 46.72% | 21264 | 2306 (10.8%) |
| G24h_2 | 43.83 | 42.36 | 41.08 | 1.28 | 46.57% | 21113 | 2250 (10.7%) |
| G24h_3 | 47.34 | 45.62 | 44.15 | 1.47 | 46.62% | 21231 | 2315 (10.9%) |
| G120h_1 | 44.37 | 42.88 | 41.84 | 1.04 | 46.51% | 20881 | 2233 (10.7%) |
| G120h_2 | 37.07 | 35.78 | 34.83 | 0.95 | 46.68% | 20413 | 2100 (10.3%) |
| G120h_3 | 39.04 | 37.28 | 36.52 | 0.77 | 46.66% | 19877 | 1986 (10.0%) |

**Table S3.** qPCR rimers used in this study.

| **Gene ID** | **Primer name** | **Sequence (5’-3’)** |
| --- | --- | --- |
| NNU_13989 | NnERFV-II1_F | ATCCCTCGCAACAGAAGTCGT |
|  | NnERFV-II1_R | GAGAAAGGCACCTGAAGGAGAA |
| NNU_05779 | NnERFV-II2_F | ACAGGGAAGCAAGGAAGATAAGAG |
|  | NnERFV-II2_R | ATAGTAACCATTGGCAGGAAACG |
| NNU_13993 | NnERFV-II3_F | TGTAGGGACGACGGGTGGTAA |
|  | NnERFV-II3_R | CCTCTTCGGCGGTGTTGTAAG |
| NNU_11724 | NnERFV-II4_F | AGGAAGCAGTGCCTAACGAGG |
|  | NnERFV-II4_R | GGTGGGAGGAGGTCATCAAAG |
| NNU_05030 | NnERFV-II5_F | CTTTCAGCCTTTGAGTCCCAGAT |
|  | NnERFV-II5_R | CCAACAACCATTCCTCCCACC |
| NNU_05776 | NnERFV-II6_F | CGAGCAAAGGAACCCGTGATA |
|  | NnERFV-II6_R | CCTCTTCGGCGGTGTTGTAAG |
| NNU_25645 | NnACS1_F | TTTCGAGTGGGTGCCATTTAC |
|  | NnACS1_R | TTACTCTTCAAGCATCTGATACCG |
| NNU_12026 | NnACS2_F | CCCAATAGAATAGTCCTCACAGC |
|  | NnACS2_R | CAATCTTGACGCCAGTCCTCC |
| NNU_13826 | NnACS3_F | TTTTAAGAAAGCACTGGTGGAA |
|  | NnACS3_R | CAAACCCTGGATAGTATGGAGC |
| NNU_16817 | NnACS4_F | TAGAAGAAGCATACGGTAGAGCG |
|  | NnACS4_R | ATTCGGACGAGGAGAAGACAG |
| NNU_10421 | NnERF1_F | AAACAGAAAGAAGAGCCCAAGACC |
|  | NnERF1_R | CGATCCACGCATCGAGAAAGC |
| NNU_07293 | NnEXP1_F | CGTTGTTGGGACGAAGTGAGA |
|  | NnEXP1_R | CTAGTGGCGGGTGATAAGGGA |
| NNU_04640 | NnEXP2_F | AGGCATCCGTTTCACCATCAA |
|  | NnEXP2_R | AGACTACGCTACGCCCATCAC |
| NNU_23652 | NnEXP3_F | CTTCCTTGGTGTTTCCCTGAT |
|  | NnEXP3_R | TGACTGTATAGATTTCCATACCCACA |
| NNU_12640 | NnPDC1_F | ATCTTCCCATCATTTGTATCGTCG |
|  | NnPDC1_R | TCAGTTCGTGAGCGTCTTCCA |
| NNU_13080 | NnPDC2_F | ACTGGGACTACACTGGATTCGTT |
|  | NnPDC2_R | ATTGGCAGCACAGACCCTTGA |
| NNU_00240 | NnPDC3_F | CAGGAGCTTCGGTGCTTTCAG |
|  | NnPDC3_R | CTGCTGCTTCCAGTCCCATTT |
| NNU_14241 | NnADH1_F | ACCCAATGGACCATGAGAAAC |
|  | NnADH1_R | CACCCACAAGAACAGCAACAC |
| NNU_14240 | NnADH2_F | GATTACTGATGGAGGAGTGGACC |
|  | NnADH2_R | TCGGAGCGTGGTTTGTAGTTT |
| NNU_16725 | NnADH3_F | AATGGAAACCCAATCAATCACT |
|  | NnADH3_R | GTTTAGCCACGTTTAGAGTAGCAC |
| NNU_19262 | NnCIPK15a_F | TCAGATCAGACAGGATGGGTTGT |
|  | NnCIPK15a_R | TCGGAACTCGCCCTTGTAAAT |
| NNU_00930 | NnCIPK15b_F | AGGAAATTGTGGCTGTGAGTGG |
|  | NnCIPK15b_R | ACCCGACCTTCAGCACCTTCT |
| NNU_19321 | NnCIPK15c_F | CGCTCGTGCTTACTTCCAACA |
|  | NnCIPK15c_R | TCGCTCCATCGTATCCCTTCT |
| NNU_23926 | Nnα-amylase1_F | TGGAAAGAAAGGTCCCTGATA |
|  | Nnα-amylase1_R | CCATCGCTCTAACTTTGTATTG |
| NNU_07806 | Nnα-amylase2_F | AAACCACCGCTGTGCCCACTA |
|  | Nnα-amylase2_R | AAGCCACTCTTTCAGATCGTTCCTC |
| NNU_07200 | Nnα-amylase3_F | GGCAGATGCTGATAAGGTTGT |
|  | Nnα-amylase3_R | TAGCCTCCTCCAGAAATGTTG |
| NNU_13572 | Nnα-amylase4_F | TGCCAATCCAAGAGTCCAAAG |
|  | Nnα-amylase4_R | TACCCATTCAACCATCTCATAACG |
| NNU_07777 | NnNR_F | GAGCGTTTCCTCGCCTTTCAT |
|  | NnNR_R | GGCTTTGTCGGAGTGGATTGC |
| NNU_11929 | NnBR1_F | ACACCCATAAGTTCCCTTCCT |
|  | NnBR1_R | AATCTAATGCCTTCCACCAGAG |
| NNU_24605 | NnPR1_F | ACAGGACTACATAAATGCCCACAA |
|  | NnPR1_R | TTTCGTTTACCCACATCTTCACAG |
| NNU_24602 | NnPR2_F | ACAGGACTACATAAATGCCCACAA |
|  | NnPR2_R | CGTTTACCCACATCTTCACAGCA |
| NNU_04043 | NnPRPL_F | TTTTCCTCGTGGGTTTGCTTG |
|  | NnPRPL_R | AGGGTTGTCTTGTGCTGTGGC |
| NNU_03160 | NnNCED1_F | ACATTCCTTCACTGCCCATCC |
|  | NnNCED1_R | TTTCCCTTCACCATTTCCTCA |
| NNU_03163 | NnNCED2_F | GGAGGAGCAAAGTTTATGAAGA |
|  | NnNCED2_R | AGTCCAACATTCCAAGGGTTT |
| NNU_23070 | NnNCED3_F | ATGCCTTCTCATCCTCTTTCTTCC |
|  | NnNCED3_R | CTGGCTTTGTCTTTGTTGTGCTCT |
| NNU_23071 | NnNCED4_F | ACACGAGTCTGGCTTTCTTTGGC |
|  | NnNCED4_R | GGCCGTCGCATTGGATTGTAG |
| NNU_02734 | NnNCED5_F | ACCACCCTCTACCCAAGACAGC |
|  | NnNCED5_R | CGATACGAACAGCGTGAACCATA |
| NNU_11066 | NnNCED6_F | CGATCAGCAGGTGGTGTTCAA |
|  | NnNCED6_R | CTCCCAAGCGTTCCAGAGGTG |
| NNU_03595 | NnCYP707A1_F | CTCTGATGTCGAGCGGATTGT |
|  | NnCYP707A1_R | TCCTTGCCTTTATTGCTTTGC |
| NNU_18754 | NnCYP707A2_F | TCTGCCACCTATCTTTCTCATC |
|  | NnCYP707A2_R | ACCATACCGTTTCTGCCTCTT |
| NNU_02459 | NnCYP707A3_F | GAACTCAAACGGTGCTACTACAT |
|  | NnCYP707A3_R | TCTTCCTTCCTCTGCCTCCTG |
| NNU_23085 | NnCYP707A4_F | CACGAGGAAGATGCCAATAAG |
|  | NnCYP707A4_R | CTCAAATCTGGAGGGATCAAA |
| NNU_13574 | NnCYP707A5_F | TCACCAAGGCGACTACCACAG |
|  | NnCYP707A5_R | CAAGCGTAACCACCATTATTTC |
| NNU_21546 | NnGA3OX1_F | GAACAGCACAAGCGGTTTACAGG |
|  | NnGA3OX1_R | CGTCGGCGGTCCATAGAGGTAG |
| NNU_26278 | NnGA3OX2_F | CCTCGCTCGAATCTCCTCCTT |
|  | NnGA3OX2_R | GCTGCCCACTTTATGTCATCCTT |
| NNU_26277 | NnGA3OX3_F | GCGACCTGCTCCAGATCCTCT |
|  | NnGA3OX3_R | CCTTGATGCCAATGTACTCTTTCC |
| NNU_21548 | NnGA3OX4_F | CATTGACCTCTGTGACCCTAAAGC |
|  | NnGA3OX4_R | CATAGCCTGTGAAACCGTCTGG |


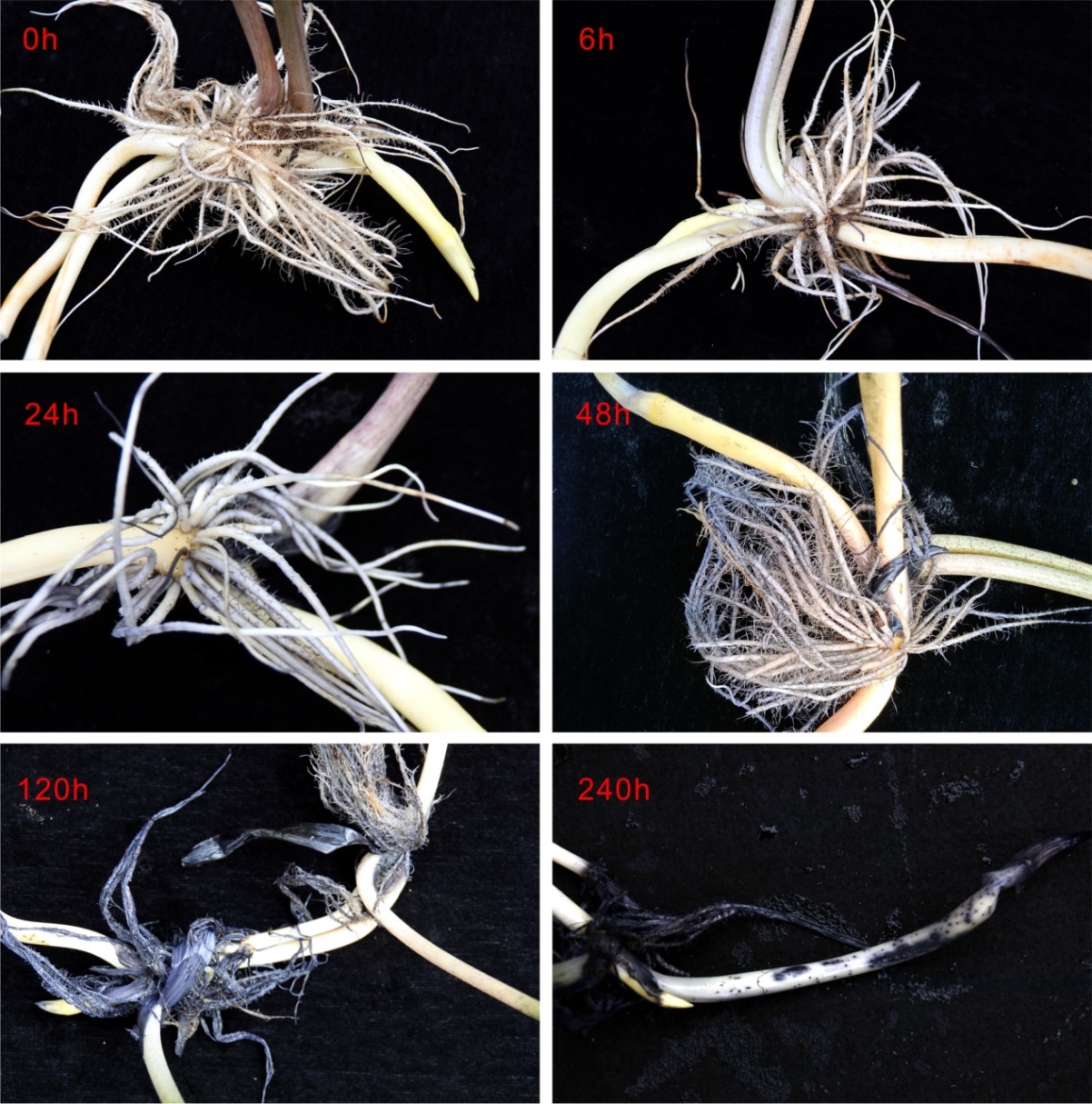


**Fig. S1** Cell death symptoms on the root and rhizome tissues of ‘Qiuxing’ under complete submergence treatment at different time points.


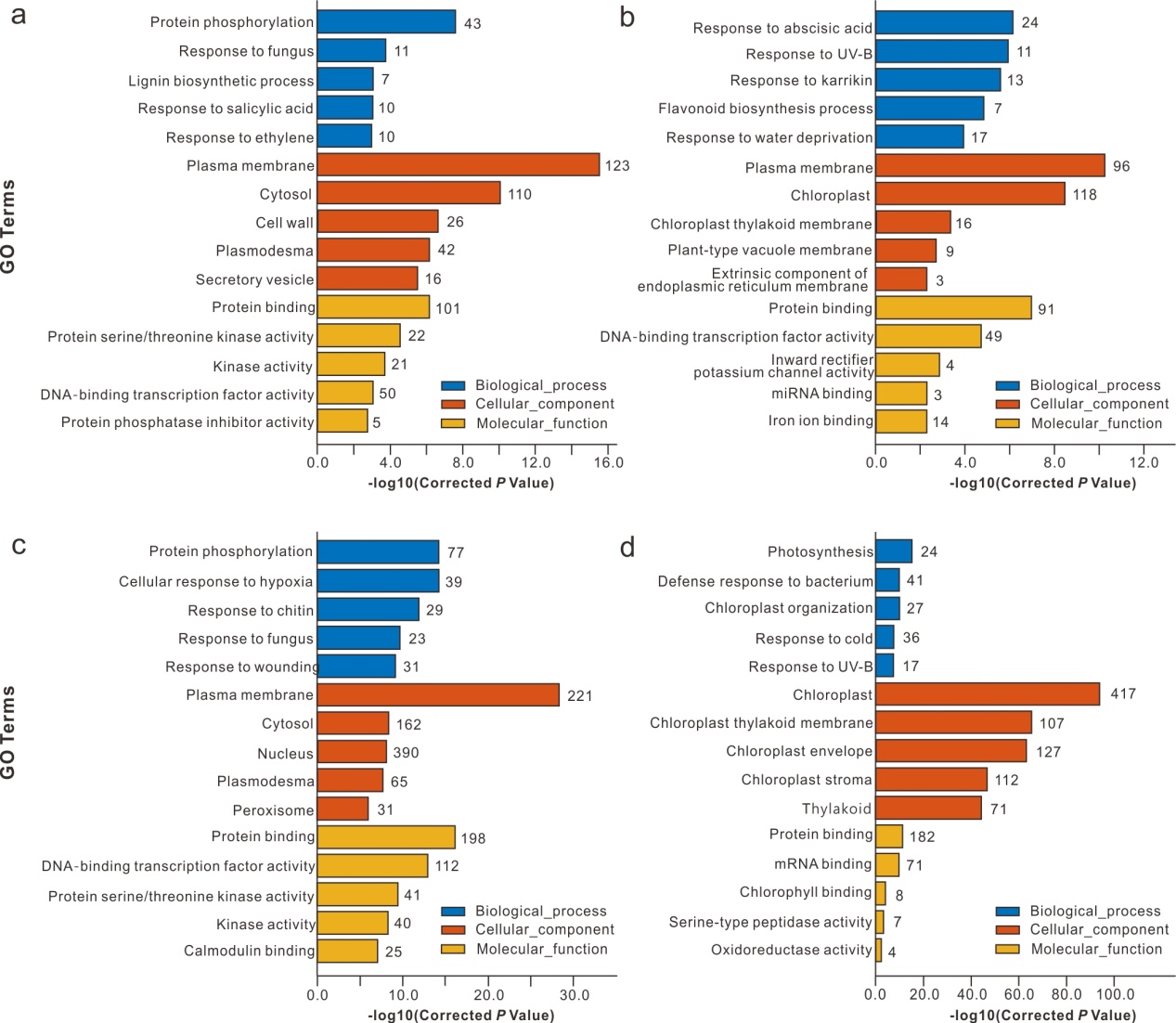


**Fig. S2** Significantly enriched Gene Ontology (GO) terms (*P* ≤ 0.05) of the common DEGs among the four submerged comparisons. (a-b) Enriched GO terms of up- and down-regulated DEGs respectively in ‘Qiuxing’ variety. (c-d) Enriched GO terms of up- and down-regulated DEGs respectively in “China Antique” variety. GO terms belong to biological processes, molecular functions, and cellular components were shown in blue, brown, and yellow, respectively.


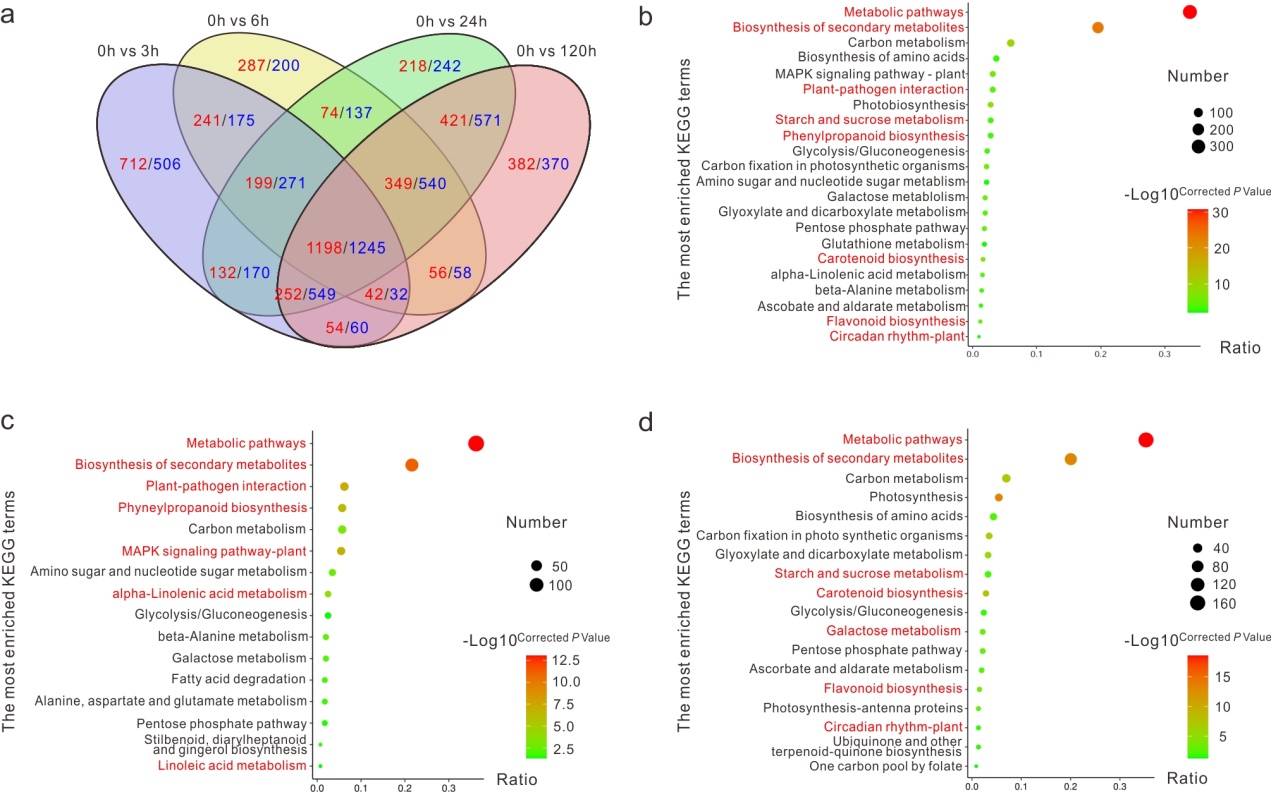


**Fig. S3** Identification of differentially expressed genes (DEGs) in submerged ‘China Antique’ variety. (a) Venn diagrams showing the numbers of DGEs in different submergence comparisons, and the commonly up- or down-regulated DEGs among submergence comparisons. Red and blue color represents the number of up- and down-regulated genes respectively. (b) Kyoto Encyclopedia of Genes and Genomes (KEGG) enrichment analysis of the 2,445 common DEGs among the 4 submerged comparisons. (c) and (d) KEGG enrichment analysis for the up-/down-regulated DEGs, respectively. Terms marked in red are those that shared both in the ‘Qiuxing’ and ‘China Antique’ varieties.


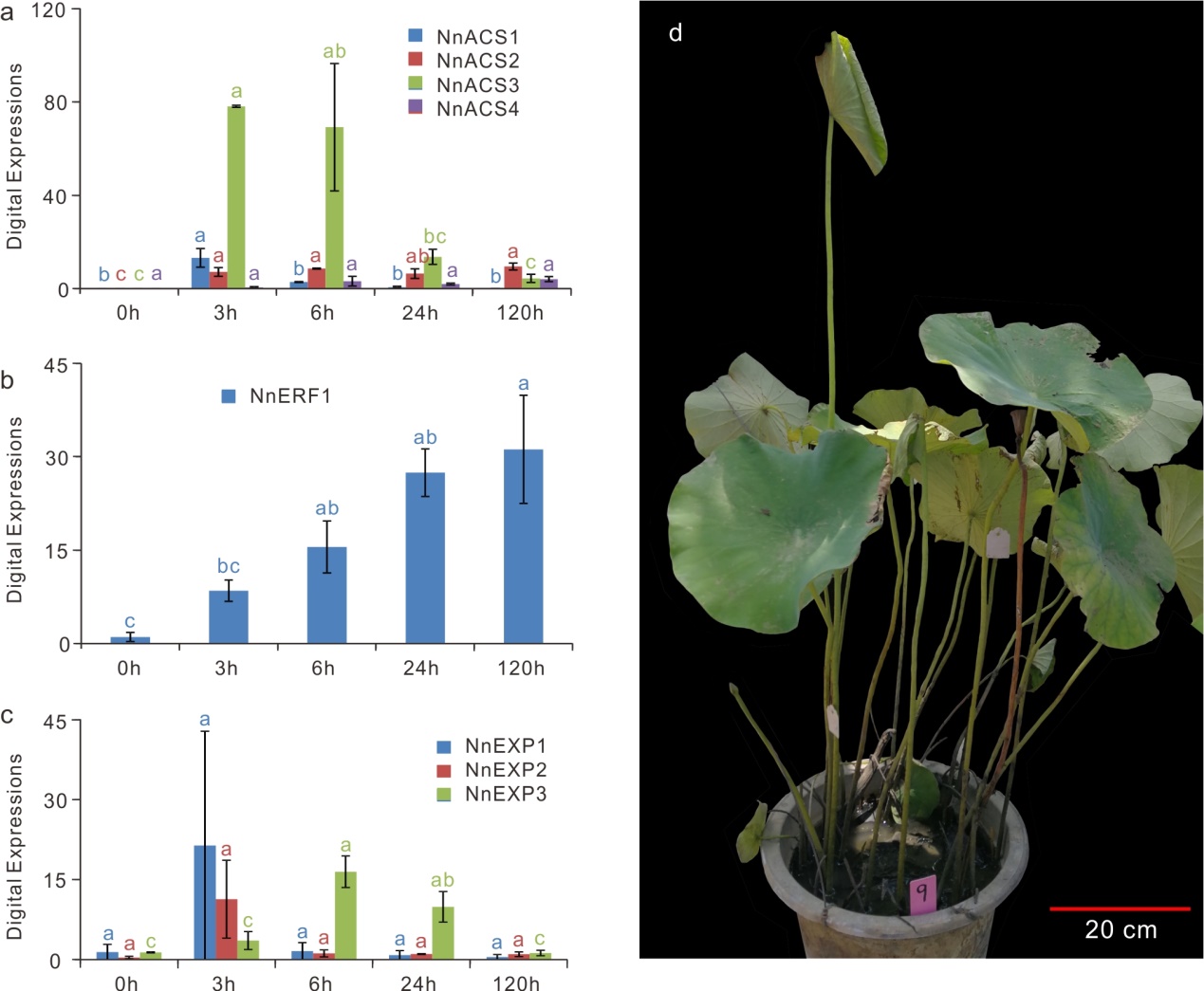


**Fig. S4** Submergence significantly enhanced petiole elongation in ‘China Antique’. Genes encoding ACC synthases (NnACSs) (a), ethylene marker protein (NnERF1) (b), and expansions (NnEXPs) (c) were significantly up-regulated. (d) Petioles of newly developed leaves were significantly elongated than those developed under aerobic conditions. Data are based on FPKM values of each gene, and error bars indicate standard error (SE) of three biological replicates. Significance of differences (*P* < 0.05) was marked with lowercase letters.


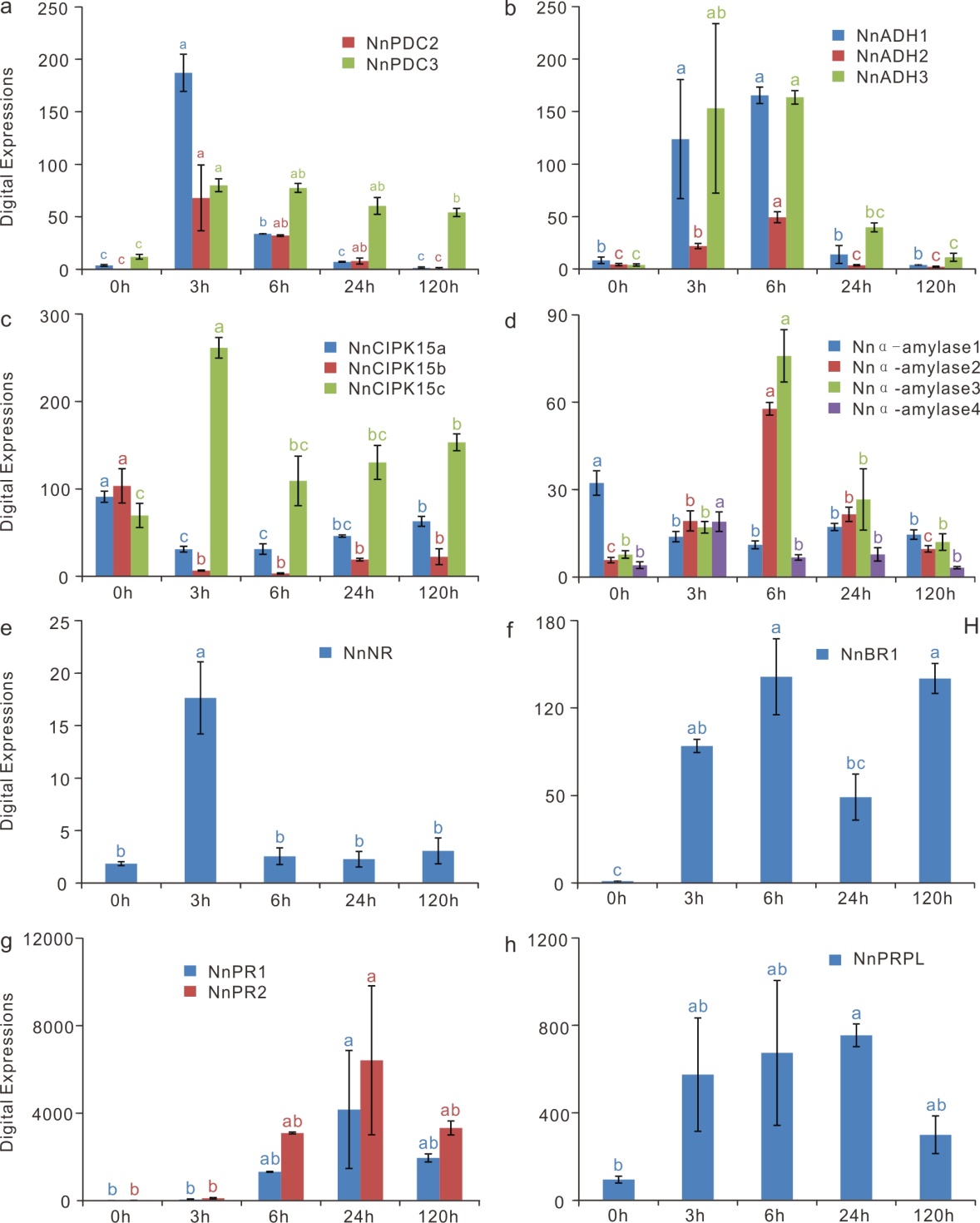


**Fig. S5** ‘China Antique’ plants increased their anaerobic fermentation and pathogen resistance under complete submergence. (a-e) Expressions of lotus genes encoding pyruvate decarboxylases (PDCs), alcohol dehydrogenases (ADHs), B-like protein-interacting protein kinase15 (CIPK15s), α-amylases, and a nitrate reductase (NR). (f-h)Expressions of lotus genes encoding a NnBRI, 2 PATHOGENESIS-RELATED GENE 1s (NnPR1s), and a pathogen-related protein-like protein (NnPRPL). Data are based on FPKM values of each gene, and the error bars indicate standard error (SE) of three biological replicates. Significance of differences (*P* < 0.05) was marked with lowercase letters.


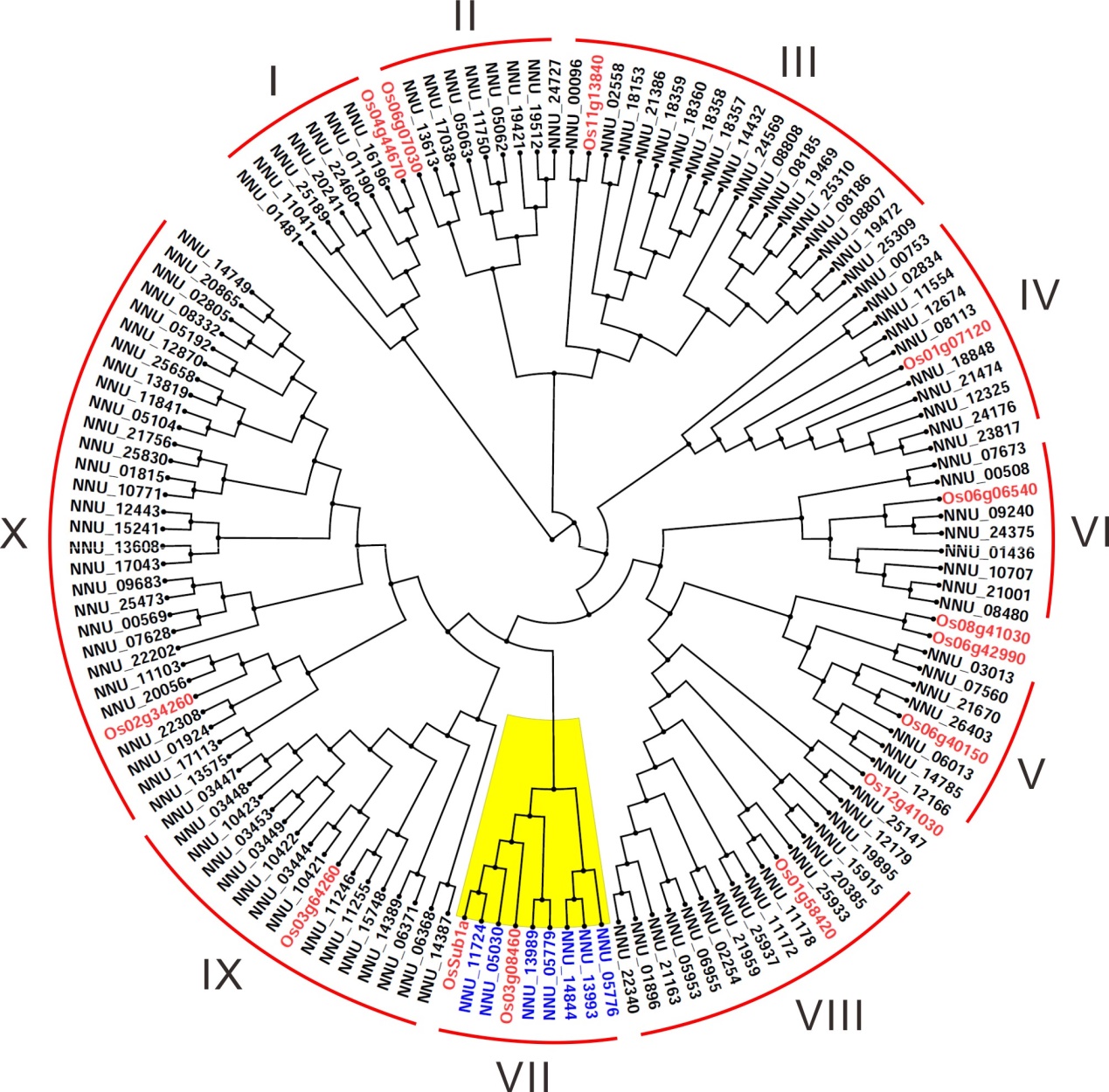


**Fig. S6** Phylogenetic tree of lotus ERF proteins. Reference genes from rice representing different ERF groups were marked in red, and lotus ERF proteins located in the ERF-VII group were marked in blue.


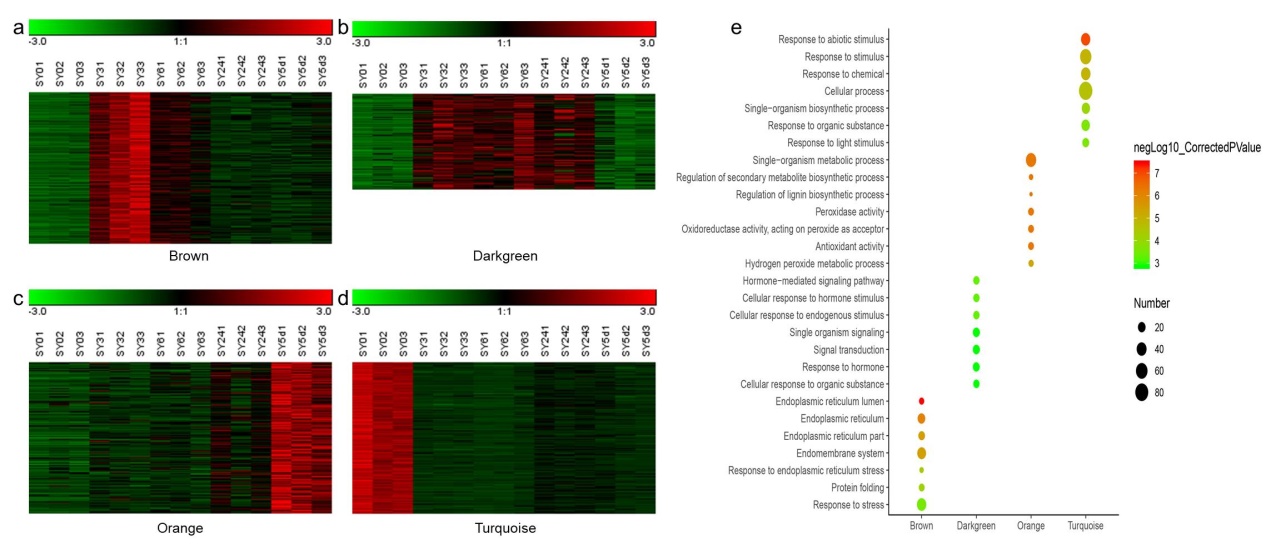


**Fig. S7** Expression patterns (a-d) and KEGG enrichment analysis (e) of DEGs in four representative modules that were identified by weighted gene co-expression network analysis (WGCNA).


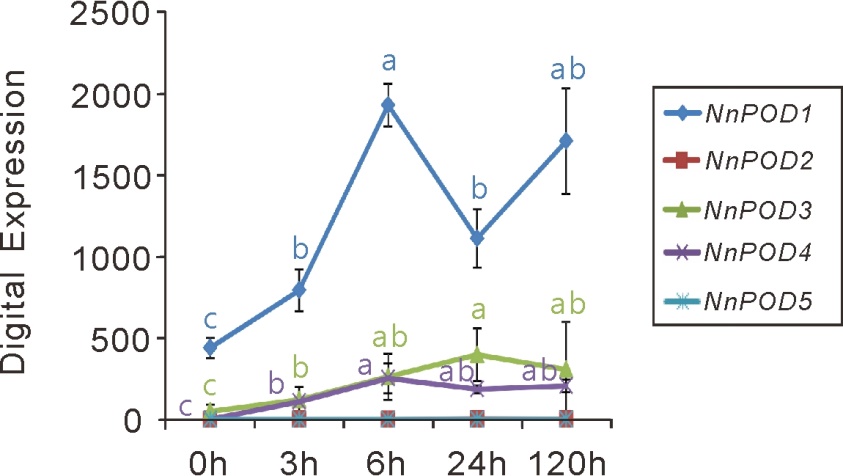


**Fig. S8** Expression of lotus peroxidases in lotus ‘Qiuxing’ under complete submergence. Data are based on FPKM values of each gene, and the error bars indicate standard error (SE) of three biological replicates. Significance of differences (*P* < 0.05) was marked with lowercase letters.
